# Elevated interleukin (IL)-6 as a predictor of disease severity among Covid-19 patients: a prospective cohort study

**DOI:** 10.1186/s12879-023-08294-w

**Published:** 2023-05-09

**Authors:** Bahram Nikkhoo, Matin Mohammadi, Sabah Hasani, Naseh Sigari, Aryan Borhani, Chia Ramezani, Arian Charajoo, Shaho Badri, Farzin Rostami, Mashala Etemadi, Khaled Rahmani

**Affiliations:** 1 Liver and Digestive Research Center, Research Institute for Health Development, Kurdistan University of Medical Sciences, Sanandaj, Iran; 2 Kurdistan University of Medical Sciences, Sanandaj, Iran; 3 Lung Diseases and Allergy Research Center, Research Institute for Health Development, Kurdistan University of Medical Sciences, Sanandaj, Iran

**Keywords:** Covid-19, SARSCoV-2, Interlukine-6, Severity

## Abstract

**Background:**

accompanied to the spreading of coronavirus disease 2019 (Covid-19) in the world, identifying factors related to the severity of the disease is one of the interests of physician and medical researchers. We hypothesized that interleukin 6 serum level is associated with severe outcome.

**Methods:**

In this longitudinal prospective cohort study we enrolled 208 confirmed COVID-19 patients who were admitted to the Tohid Hospital (Sanandaj, Iran). Patients were classified into two groups based on IL-6 value in the first day of admission, elevated (n = 107) or not elevated/normal (n = 101), and followed until the occurrence of final outcome (death or discharge from the hospital). Data were analyzed using univariate methods, Chi-squared and independent two sample T test. The relationship between the independent variables and our interesting outcomes were investigated by multiple linear and penalized logistic regression modeling.

**Results:**

A total of 208 patients, 51% female and mean age 53.6 ± 16.3 years, including 107 elevated and 101 non-elevated IL-6 patients, were followed. No significant difference was observed between the two groups in demographic and clinical characteristics. Although not significant, logistic regression results showed that the chance of death occurrence among patients with elevated IL-6 are 3.91 times higher. According to the multiple linear regression modeling, elevated IL-6 significantly increased the duration of hospital stay (P = 0.02). Frequency of ICU admission (P = 0.04) and mean of ICU stay (P = 0.8) are also higher in elevated IL-6 group.

**Conclusion:**

This study revealed that elevated IL-6 is significantly related to prolongation of hospital stay in Covid-19 patients. Although not significant, the occurrence of death among patients who had increased IL-6 in the time of admission was higher than patients with normal or lower serum levels of IL-6.

## Background

China had reported to the World Health organization (WHO) on 31st December 2019 about the outbreak of a novel corona virus (n-CoV) causing pneumonia in adults in the city of Wuhan [1]. Despite fragmented international efforts to contain the spread, SARS-CoV2 has spread to more than 213 countries, since its formal identification [2]. The Covid-19 pandemic as a challenging health issue during the recent years has about 2.3% crude mortality rate. This infection has unpredictable heterogeneous disease course. Although the most confirmed cases are mild, with cold-like symptoms to mild pneumonia, near to 14% of patients experience the severe conditions including serious pneumonia and breath shortness. It should be noted that in about 5% of cases (critically ill patients) disease can lead to developed respiratory failure, septic shock or/and multi-organ failure which is potentially life-threatening [3].

According to the laboratory study and pathological examination, SARS-CoV-2 as a cytopathic disease can have caused the first damage to the lung [4]. Accompanied viral proliferation and amplification, host immune response usually activated to clear the virus and cure the patients although some cases had more severe disease development like multiple organ dysfunction syndrome (MODS) that is still unknown [5]. It seems cytokine storm has an important role in the pathogenesis of severe cases of COVID-19. Based on exist evidence, cytokines are central to the pathophysiology of COVID-19; while some of them are beneficial, others appear detrimental such as interleukin-6 particularly in the context of the cytokine storm [6]. In fact, various infectious or non-infectious disease can be resulted in cytokine storms and multiple organ damages [7]. An overproduction of IL-6 and dysregulation of the IL-6 signaling pathways can result in inflammation and play an important role in the human cytokine network [8].

In a study conducted by Luo et al., efficacy of tocilizumab (antibody against IL-6) in patients with COVID‐19 has been evaluated. They demonstrated that use of this medication, particularly in patients at risk of developing chemokine storm secondary to COVID‐19 is encouraging [9]. IL-6 signaling plays a pivotal role in controlling the differentiation and activation of T lymphocytes by inducing the Jak/STAT-3 and the Ras/Erk/C/EBP pathways. In particular, IL-6 modulates the resistance of T cells against apoptosis, induces activation of T helper cells and controls the balance between regulatory T cells and Th17 cells [10].

There are several studies that declared IL-6 blockade is a promising strategy for COVID-induced CRS (Cytokine Release Syndrome) and elevated IL-6 levels were consistently reported in them and IL-6 might serve as a predictive biomarker for disease severity [11]. Another large retrospective cohort study found that IL-6 levels were correlated with mortality in patients with COVID-19 [12]. However, some previous studies have shown an association between IL-6 levels and COVID-19 disease, there is a study that did not show any evidence of an association between the two [13]. According to the study of Yong Xiong et al., relatively lower expression levels of IL-6 receptor were observed in bronchoalveolar lavage fluid (BALF) of patients with COVID-19 in comparison with healthy individual, and there are no significant differences in peripheral blood mononuclear cells (PBMC), suggests that IL-6/IL6R axis of cells in BALF and PBMC might not be involved in the COVID-19 pathophysiology [13]. Additionally, most of the previously conducted research have retrospective design without attention to the control of potential effects of confounding factors such as age [11, 14, 15]. In this longitudinal study we aimed to investigate the association between Il-6 and disease severity of COVID-19.

## Methods

In this longitudinal cohort study, we initially included 210 confirmed COVID-19 patients, Kurdish ethnic, with laboratory (PCR) confirmation of covid_19, who were admitted to the Tohid Hospital in Sanandaj, the center of Kurdistan province, Iran, between September 11 and December 28, 2021. The time frame of conducting this study coincided with the prevalence of Delta strain, highly contagious SARS-CoV-2 virus strain, in Kurdistan province. In addition to definite diagnosis of COVID-19, age ≥ 15, and not pregnancy were our inclusion criteria for study subjects. Patients younger than 15, patients who had missing data on IL-6 levels or other study variables, patients who had positive pregnancy test during the data collection were excluded from the study. Two patients were excluded from the study due to referral to another treatment center outside the province during the follow-up, and 208 cases were remained that their data entered to the analysis.

### Data collection, follow up and outcomes

Detailed patient information, including demographic characteristics, result of SARS-CoV-2 nucleic acid detection, history of comorbidities and drug uses, laboratory findings, computed tomography (CT) findings, length of hospital stay, oxygen support, and illness severity on admission was collected from the patient records stored in the hospital information management system and entered into a customized Excel form. Patients were followed during hospitalization and those who were died or transferred to ICU or another hospital inside the Sanandaj city were checked and followed up. In first day of admission, for each patient, serum sample was obtained in the first day of hospitalization and is sent to the central pathobiology laboratory to assess the IL-6. In the central laboratory, IL-6 values were measured using Electrochemiluminescence methods in COBAS e 411 analyzer. Based on the IL-6 values patients were divided into two groups; those with IL-6 higher than cut-off or elevated IL-6 (exposed group) and lower than cut-off or not elevated IL-6 (unexposed group). The patients of both groups were followed up until the occurrence of final outcome (death or remission and discharge from the hospital).

In addition to the final/main outcome (death or recovery and discharge from the hospital), duration of hospital stays due to Covid-19 is another main outcome that measured for the patients. Admission in intensive care unit (ICU) and duration of ICU stay, were secondary interesting outcomes that were evaluated and compared between the two groups.

### Ethical consideration

The study protocol was reviewed and approved by the Research Ethics Committee of Kurdistan University of Medical Sciences (ethics code: IR.MUK.REC.1400.067). before the data collection the research objectives and study protocol were explained for each of the participants and written informed consent was obtained.

### Statistical analysis

Statistical analyses were conducted in Stata Statistical Software version 16 (Stata Corp. 2019. Stata Statistical Software: Release 16. College Station, TX: StataCorp LLC). Data were summarized using descriptive statistics such as mean, standard deviation (SD), frequency and percent. Normality assumption of quantitative data was checked by Kolmogorov–Smirnov. Normally distributed variables were expressed as mean ± SD and were analyzed by independent samples t-test. Multiple linear and penalized Logistic regression was used to evaluate the relationship between independent variables and two our interesting outcomes including hospital stay and death resulting from covid-19, respectively.

## Results

A total of 208 covid-19 patients, 101 patients in the increased IL-6 group and 107 patients in the control group or non-increased IL-6 group, at the time of admission were investigated. demographic and clinical characteristics of study participants are detailed in Table 1.

**Table 1:** Demographic and clinical characteristics of study participants

		Elevated IL-6 group, n = 101	Normal or non-elevated IL-6 group, n = 107	P value
Age (year), mean ± SD		55.57$\pm$17.12	51.72$\pm$15.29	0.09
BMI, mean ± SD		27.50$\pm$4.27	27.90$\pm$4.73	0.52
Sex, n (%)	Female	45 (42.5)	61 (57.5)	0.07
	Male	56 (54.9)	46 (45.1)	
HTN, n (%)	No	73 (47.1)	82 (52.9)	0.47
	Yes	28 (52.8)	25 (47.2)	
DM, n (%)	No	81 (48.8)	85 (51.2)	0.89
	Yes	20 (47.6)	22 (52.4)	
COPD, n (%)	No	100 (49.5)	102 (50.5)	0.21
	Yes	1 (16.7)	5 (83.5)	
CVD, n (%)	No	92 (48.4)	98 (51.6)	0.90
	Yes	9 (50)	9 (50)	
Cancer, n (%)	No	99 (49.7)	100 (50.3)	0.17
	Yes	2 (22.2)	7 (77.8)	
CRF, n (%)	No	96 (48.0)	104 (52.0)	0.49
	Yes	5 (62.5)	3 (37.5)	
Autoimmune disease, n (%)	No	98 (48.0)	106 (52.0)	0.29
	Yes	3 (75.0)	1 (25.0)	
RA, n (%)	No	92 (47.7)	101 (52.3)	0.36
	Yes	9 (60.0)	6 (40.0)	
Dyslipidemia, n (%)	No	96 (49.2)	99 (50.8)	0.45
	Yes	5 (38.5)	8 (61.5)	
Asthma, n (%)	No	95 (49.2)	98 (50.8)	0.50
	Yes	6 (40.0)	9 (60.0)	
SBP, mean ± SD		132.63$\pm$19.83	131.32$\pm$17.10	0.61
DBP, mean ± SD		81.59$\pm$14.41	82.78$\pm$9.87	0.49
T, mean ± SD		36.83$\pm$0.69	36.60$\pm$0.48	0.99
RR, mean ± SD		20.67$\pm$7.16	20.58$\pm$7.20	0.93
h, mean ± SD		92.25$\pm$18.32	92.70$\pm$14.11	0.84
O_2_ SAT, mean ± SD		89.80$\pm$6.28	91.60$\pm$4.96	0.23
WBC, mean ± SD		5.99$\pm$2.93	5.51$\pm$2.43	0.21
Neutrophil, mean ± SD		78.47$\pm$8.66	77.64$\pm$9.67	0.54
NLR, mean ± SD		4.63$\pm$2.74	4.94$\pm$5.24	0.63
Platelet, mean ± SD		186.55$\pm$71.35	198.05$\pm$90.60	0.32
AST, mean ± SD		41.87$\pm$24.68	43.66$\pm$40.38	0.71
ALT, mean ± SD		39.37$\pm$35.10	40.95$\pm$36.13	0.75
CRP, mean ± SD		73.91$\pm$54.30	74.97$\pm$49.92	0.93

**BMI**: Body mass index; **HTN**: Hypertension; **DM**: Diabetes mellitus; **COPD**: Chronic obstructive pulmonary disease; **CVD**: Cardiovascular disease; **CRF**: Chronic renal failure; **RA**: Rheumatoid arthritis; **SBP**: Systolic blood pressure; **DBP**: Diastolic blood pressure; **T**: Temperature; **RR**: Respiratory rate; **HR**: Heart rate; **NLR**: Neutrophil to lymphocyte ratio; **AST**: Aspartate aminotransferase; **ALT**: alanine transaminase; **CRP**: C-reactive protein

As shown in Table 1, no significant differences observed between the two groups regarding demographic and clinical characteristics. Table 2 indicates the mean of hospitalization days and frequency of ICU admission and death as main outcome of the study in the study groups.

**Table 2:** Mean of hospital stay and frequency of ICU admission and death in studied patients

		Elevated IL-6 group, n = 101	Normal or non-elevated IL-6 group, n = 107	P value
Hospitalization duration		10.00 ± 8.67	6.69 ± 4.56	0.001
ICU stay duration		8.88 ± 7.94	7.50 ± 6.36	0.82
ICU admission, n (%)	No	93 (92.1)	105 (98.1)	0.041
	yes	8 (7.9)	2 (1.9)	
Death, n (%)	No	93 (92.1)	105 (98.1)	0.041
	yes	8 (7.9)	2 (1.9)	

As indicated in Table 2, the mean duration of hospital stay in patients with increased IL-6 was significantly 3.3 days higher than those who have no increased IL-6. Frequency of ICU admission and death in patients with increased IL-6 (7.9%) was also more than no-increased IL-6 group (0.04). although not significant but the mean of ICU stays in increased IL-6 group approximately 1.4 days higher than no-increased IL-6 group (p = 0.82). in Table 3, univariate and multivariate analysis to assess the potential association between death and studied variables are summarized.

**Table 3:** Penalized logistic regression results of relationship between death and studied factors

	Un-adjusted		Adjusted	
	OR (95%CI)	P value	OR (95%CI)	P value
Age	1.08 (1.03–1.14)	0.002	1.07 (1.01–1.13)	0.03
Sex, male	1.59 (0.44–5.82)	0.48	1.19 (0.28–5.06)	0.81
BMI	0.87 (0.77–0.99)	0.038	0.91 (0.77–1.09)	0.30
Temperature	0.87 (0.28–2.68)	0.81	0.71 (0.14–3.63)	0.68
HTN	0.72 (0.15–3.50)	0.68	0.26 (0.04–1.51)	0.13
Diabetes	4.35 (1.20–15.81)	0.021	4.17 (0.91–19.07)	0.07
Increased IL-6, cases	4.52 (0.93–21.80)	0.051	3.91 (0.68–22.44)	0.12

The results of multiple penalized logistic regression modeling to assess the relationship between death from covid-19 and independent variables such as increased IL-6 demonstrated that age is only significant factor for death (OR = 1.07, P = 0.03). Albeit not significant but chance of death in Increased IL-6 patients 3.91 times was higher than non-increased IL-6 group. Another important factor was diabetes so that patients with type-2 diabetes had higher risk of death in comparison with those who have not (OR = 4.17, P = 0.07).

**Table 4:** Multiple linear regression results of relationship between studied variables and average length of stay in hospitals

	Coef. (95%CI)	P value
Age	0.057 (-0.001–0.115)	0.05
Sex, male	0.785 (-0.934–2.505)	0.37
BMI	0.001 (− 0.004–0.006)	0.79
Temperature	1.629 (0.213–3.045)	0.02
HTN	-0.963 (-3.156–1.229)	0.39
Diabetes	-0.211 (-2.469–2.047)	0.85
Increased IL-6, cases	2.104 (0.390–3.817)	0.01

R-squared = 0.12, p = 0.002

Another main interesting outcome was hospital stay. The results of multiple linear regression modeling (Table 4) showed that increased temperature (P = 0.02) and Increased IL-6 (P = 0.01) are significantly related to the length of hospital stay. Although not significant, increased age was another factor that related which has been associated with an increase in the duration of hospitalization.

## Discussion

This study is conducted to evaluate the disease severity based on values of IL-6 among covid-19 patients. The analysis demonstrated that elevated IL-6 was related to the poorer outcome so that duration of hospital stay and mortality were higher in patients with elevated IL-6 at the onset of hospital admission. According to the results of any previous studies, IL-6 as a potent proinflammatory cytokine can stimulate c-reactive protein (CRP) and has been introduced as a trigger of the cytokine storm in COVID-19 [16–20]. In this study we evaluate the relationship between serum level of IL-6 in the time of admission and disease severity among Covid-19 patients. Our results indicated that elevated IL-6 are associated with longer hospital stay and higher mortality that consistent with the results of previous studies. Keddie et al., showed that IL-6,CRP and interleukin 10 (IL-10) are strongly correlated with the illness severity [21]. Zulvikar Syambani Ulhaq et al. also indicated that IL-6 has major importance because of its pleiotropic effects and circulating IL-6 serum levels can be closely linked to the severity of infection in Covid-19 [22]. Association between IL-6 increasing and respiratory dysfunction has been addressed previously which imply a possible shared mechanism of cytokine-mediated lung damage resulted from SARS-CoV-2 infection [23]. Additionally, highly pathogenic SARS-CoV-2 can be associated with rapid replication of virus and a tendency to involve the lower respiratory system, resulting in an elevated response of IL-6-induced severe respiratory distress. Thus, these evidences suggest that immediately initial evaluation after hospitalization and serial measurement of IL-6 may be has an important role in assessing worsening clinical features and disease progression among Covid-19 patients. It should be noted that in addition to the association between IL-6 and Covid-19, increasing in IL-6 level has been proven to be a good biomarker for disease severity in other viral infections such as hepatitis B virus (HBV) [24].

In general, inflammation is closely associated with the covid-19 severity. Jing Gong et al. in a retrospective study have shown that some inflammation-related parameters such as tumor necrosis factor α (TNFα), IL-6, interleukin-2 receptor (IL2R), interleukin-8 (IL-8), interleukin-10 (IL-10), CRP, ferroprotein, procalcitonin, white cell counts (WBC), lymphocyte counts (LC), neutrophil count (NC) and eosinophil counts (EC) are correlated to severity of COVID-19 [25].

In addition to the association between the increased IL-6 level and disease severity, the other factor which was related to the outcome in our data modeling was age so that the frequency of death and length of hospital stay increased with age. This finding is consistent with the studies of Jing Gong [25], Huang [26], Zhou [27], Wang [28] and Chen [29]. In a study conducted by Mohamed El-Shabrawy et al., indicated that C-reactive protein/albumin ratio along with interleukin 6, is another biomarker of disease severity [30], although we did not examine this biomarker in the present study.

In this study we investigated the association between disease severity and some demographic and clinical variables such as diabetes mellitus, hypertension, BMI, age and gender. According to a review study, elderly age, male gender, preexisting hypertension, diabetes, obesity, COPD, tumor, immunodeficiencies, pregnancy, thromboembolism, coagulation disorders, leukocytosis, lymphopenia, eosinopenia, elevated serum levels of D-dimer, LDH, AST and ALT, BUN and creatine, cTnI, CRP, PCT, IL-6, IL-1β, KL-6, ferritin, higher CT pneumonia score, high number of affected pulmonary lobes, and smoking are major risk factors of severe clinical course and outcomes in patients with COVID-19 [31]. Indeed, assessment of all potential related factors require further studies with higher sample size and multicenter design.

Our study has some limitations. First, the number of two study groups was small due to the single center design of the study. Second, clinical data and some inflammatory response markers such as C-reactive protein (CRP), erythrocyte sedimentation rate (ESR) and plasma viscosity (PV) were limited. Lacking of sufficient data such as date of onset of symptoms to conduct the cox regression model based on the time until the desired event (death) is another limitation of the study. Based on the review of the literature, most of the previous studies that investigated the role of IL-6 in the severity of covid-19 infection were done retrospectively, and the longitudinal and being straightforward design of the present study is considered to be its most important strength. Although, a few studies have been done on Iranian covid-19 patients previously [32, 33], the current research is a first report among Kurdish population in Iran.

## Conclusion

In conclusion, based on our data, serum levels of IL-6 are significantly associated with the Covid-19 severity as independent factor to predict outcome risk. This study revealed that increased IL-6 is significantly related to prolongation of hospital stay in Covid-19 patients. Although not significant, the occurrence of death among patients who had increased IL-6 in the time of admission was higher than patients with normal or lower serum levels of IL-6.

## Data Availability

The datasets used and/or analyzed during the current study are available from the corresponding author on reasonable request.

## References

[1] Ciotti M et al. *The COVID-19 pandemic* 2020. 57(6): p. 365–388. 10.1080/10408363.2020.1783198 32645276

[2] Hiscott J (2020). The global impact of the coronavirus pandemic. Cytokine Growth Factor Rev.

[3] Team EJCCw. *The epidemiological characteristics of an outbreak of 2019 novel coronavirus diseases (COVID-19)—China, 2020*. 2020. 2(8): p. 113. PMC8392929 34594836

[4] Xu Z (2020). Pathological findings of COVID-19 associated with acute respiratory distress syndrome. The Lancet respiratory medicine.

[5] Yang X et al. *Clinical course and outcomes of critically ill patients with SARS-CoV-2 pneumonia in Wuhan, China: a single-centered, retrospective, observational study*. 2020. 8(5): p. 475–81. 10.1016/S2213-2600(20)30079-5 PMC7102538 32105632

[6] Jamilloux Y (2020). Should we stimulate or suppress immune responses in COVID-19? Cytokine and anti-cytokine interventions. Autoimmun rev.

[7] Gu Y (2019). Role of the innate cytokine storm induced by the influenza a virus. Viral Immunol.

[8] Uciechowski P, Dempke WC (2020). Interleukin-6: a masterplayer in the cytokine network. Oncology.

[9] Luo P et al. *March 2020, posting date. Tocilizumab treatment in COVID-19: a single center experience*. J Med Virol doi.10. 10.1002/jmv.25801 PMC7262125 32253759

[10] Neurath MF, Finotto S (2011). IL-6 signaling in autoimmunity, chronic inflammation and inflammation-associated cancer. Cytokine Growth Factor Rev.

[11] Zhu J et al. *Elevated interleukin-6 is associated with severity of COVID-19: a meta-analysis*. 2021. 93(1): p. 35. 10.1002/jmv.26085 PMC7283812 32470146

[12] Liu B (2020). Can we use interleukin-6 (IL-6) blockade for coronavirus disease 2019 (COVID-19)-induced cytokine release syndrome (CRS). J Autoimmun.

[13] Xiong Y et al. *Transcriptomic characteristics of bronchoalveolar lavage fluid and peripheral blood mononuclear cells in COVID-19 patients*. 2020. 9(1): p. 761–70. 10.1080/22221751.2020.1747363 PMC7170362 32228226

[14] Coomes EA. and H.J.R.i.m.v. Haghbayan, *Interleukin-6 in COVID-19: a systematic review and meta-analysis* 2020. 30(6): p. 1–9. 10.1002/rmv.2141 PMC7460877 32845568

[15] Ulhaq ZS. and G.V.J.M.e.m.i. Soraya, *Interleukin-6 Is a Potential Biomarker of COVID-19 Progression: Evidence from a Meta-Analysis* 2020. 3562887(10.2139). 10.1016/j.medmal.2020.04.002 PMC7129451 32259560

[16] Chen X, et al. Detectable serum SARS-CoV-2 viral load (RNAaemia) is closely correlated with drastically elevated interleukin 6 (IL-6) level in critically ill COVID-19 patients. Clinical infectious diseases; 2020. 10.1093/cid/ciaa449 PMC7184354 32301997

[17] Coomes EA, Haghbayan H (2020). Interleukin-6 in COVID‐19: a systematic review and meta‐analysis. Rev Med Virol.

[18] Herold T (2020). Elevated levels of IL-6 and CRP predict the need for mechanical ventilation in COVID-19. J Allergy Clin Immunol.

[19] Lagunas-Rangel FA. and V.J.J.o.m.v. Chávez‐Valencia, *High IL-6/IFN-γ ratio could be associated with severe disease in COVID-19 patients*. 2020. 92(10): p. 1789. 10.1002/jmv.25900 PMC7262117 32297995

[20] Liu F (2020). Prognostic value of interleukin-6, C-reactive protein, and procalcitonin in patients with COVID-19. J Clin Virol.

[21] Keddie S (2020). Laboratory biomarkers associated with COVID-19 severity and management. Clin Immunol.

[22] Ulhaq ZS, Soraya GV (2020). Interleukin-6 as a potential biomarker of COVID-19 progression. Med et maladies infectieuses.

[23] Bourgonje AR et al. *Angiotensin-converting enzyme 2 (ACE2), SARS-CoV-2 and the pathophysiology of coronavirus disease 2019 (COVID-19)*. 2020. 251(3): p. 228–48. 10.1002/path.5471 PMC7276767 32418199

[24] Xia C (2015). Involvement of interleukin 6 in hepatitis B viral infection. Cell Physiol Biochem.

[25] Gong J (2020). Correlation analysis between disease severity and inflammation-related parameters in patients with COVID-19: a retrospective study. BMC Infect Dis.

[26] Huang C (2020). Clinical features of patients infected with 2019 novel coronavirus in Wuhan, China. The lancet.

[27] Zhou F (2020). Clinical course and risk factors for mortality of adult inpatients with COVID-19 in Wuhan, China: a retrospective cohort study. The lancet.

[28] Wang D (2020). Clinical characteristics of 138 hospitalized patients with 2019 novel coronavirus–infected pneumonia in Wuhan. China.

[29] Chen T et al. *Clinical characteristics of 113 deceased patients with coronavirus disease 2019: retrospective study* 2020. 368. 10.1136/bmj.m1091 PMC7190011 32217556

[30] El-Shabrawy M et al. *Interleukin-6 and C-reactive protein/albumin ratio as predictors of COVID-19 severity and mortality*. 2021. 15: p. 1–7.

[31] Gao Yd et al. *Risk factors for severe and critically ill COVID-19 patients: a review*. 2021. 76(2): p. 428–55. 10.1111/all.14657 33185910

[32] Ghazanfari T et al. *Interpretation of Hematological, Biochemical, and Immunological Findings of COVID-19 Disease: Biomarkers Associated with Severity and Mortality* 2021: p. 46–66. 10.18502/ijaai.v20i1.5412 33639632

[33] Rostamian A et al. *Interleukin-6 as a Potential Predictor of COVID-19 disease severity in hospitalized patients and its association with clinical laboratory routine tests*. 2020. 3(1): p. 29–36.

